# Transparency of clinical practice guideline funding: a cross-sectional analysis of the German AWMF registry

**DOI:** 10.1186/s12910-023-00913-0

**Published:** 2023-05-19

**Authors:** Hendrik Napierala, Angela Schuster, Sabine Gehrke-Beck, Christoph Heintze

**Affiliations:** Institute of General Practice and Family Medicine, Charité – Universitätsmedizin Berlin, corporate member of Freie Universität Berlin, Humboldt Universität zu Berlin, Charitéplatz 1, 10117 Berlin, Germany

**Keywords:** Meta research, Guideline development, Clinical practice guidelines, Transparency, Guideline funding, AWMF, DELBI, Trust

## Abstract

**Background:**

While reporting of individual conflicts of interest is formalised, it is unclear to what extent the funding of clinical practice guidelines (CPGs) is formally reported. The aim of this study is to explore the accuracy and comprehensiveness of reporting on funding in German CPGs.

**Methods:**

We searched for CPGs in the registry of the Association of the Scientific Medical Societies in Germany in July 2020. Information on guideline funding was categorised by two reviewers independently and discrepancies were clarified by discussion with a third reviewer. Accuracy and comprehensiveness of reporting on funding was assessed using the German Instrument for Methodological Guideline Appraisal (DELBI).

**Results:**

We included 507 CPGs published between 2015 and 2020 in the main analysis. 23/507 (4.5%) of the CPGs achieved the highest DELBI score by including information on funding sources, expenses and the amount of funding provided, as well as a statement on the independence of the guideline authors from the funding institution(s). CPGs with more rigorous methodological requirements (systematic review of the literature and/or structured consensus-building) received higher DELBI scores.

**Conclusion:**

German CPGs do not communicate their funding transparently. Transparency of CPG funding could be achieved by making it mandatory to publish information for all guidelines. For that purpose, a standardised form and guidance should be developed.

**Supplementary Information:**

The online version contains supplementary material available at 10.1186/s12910-023-00913-0.

## Background

Clinical practice guidelines (CPGs) are *„systematically developed statements to assist practitioner and patient decisions about appropriate health care for specific clinical circumstances“* [1]. Comparable to CPG development in many other countries, in Germany they are mainly developed by scientific medical societies. The development process is coordinated by the Association of the Scientific Medical Societies (AWMF). The AWMF consists of organisations that promote scientific work focused on medicine and related disciplines [2]. The majority of the members must be clinically active physicians, dentists or university graduates involved in academic research. Most CPGs developed through the AWMF involve a diverse field of groups and experts, including patient representatives and adjacent professions (e.g., physiotherapists, midwives, pharmacists). The quality of CPGs rests on two pillars: the evidence base and the consensus-building process. In Germany, the AWMF sets standards for the development of CPGs that are mandatory for all organisations developing and publishing CPGs through the AWMF [3]. CPGs are regularly updated on the AWMF homepage [2]. The AWMF grades guidelines based on their methodological rigour [4]:

S1: A representative expert group develops a guidance in an informal consensus-building process,

S2: Guidelines are based either on a systematic analysis of the scientific evidence (S2e) or on the structured consensus of a representative committee (S2k),

S3: Guidelines use both a systematic analysis of the available evidence and a structured consensus-building process.

CPGs have a broad visibility and can influence the decision making of physicians. However, CPGs can only maintain a high quality, if they are created with sufficient financial support (e.g., for the systematic literature search, consensus conferences) [5]. External funding of both the guideline development directly and of the guideline producing organisations indirectly may have an impact on the quality of guidelines [6]. In contrast to individual COI, where a recent Cochrane review suggests that financial COI are associated with favorable recommendations of drugs and devices in CPGs [7], there is limited evidence that funding of CPGs influences guideline recommendations [8, 9].

The AWMF Guidance Manual states that CPGs influenced by third party funding cannot be registered with the AWMF [3] and recommends the AGREE-II tool for guideline development [10]. However, currently there is no transparent reporting system for funding sources and expenses. This contrasts to the reporting of individual conflicts of interests (COI) in CPGs published by the AWMF, where strict and transparent guidelines are in place. These do not only require transparent COI statements from all guideline authors but also active measures to reduce the potential influence of individual COIs (e.g., abstentions during certain voting procedures or removal from the development process). Nevertheless, there is evidence of inadequate disclosure of COI [11] and stricter measures might be necessary to mitigate this problem (e.g., a “Physician Payments Sunshine Act for Germany”) [12].

Interestingly, Campsall et al. showed that only 1% (4/290) of the studied CPGs in the United States disclosed financial relationships between the scientific medical societies and biomedical companies, although 63% (60/95) of the organizations reported receiving funds from biomedical companies on their website or in response to a survey [13]. Elder et al. found that 14 (93%) of the 15 organisations disclosed funding sources from the industry. However, none disclosed this information in the CPGs [14].

Even though Campsall et al. and Elder et al. highlighted this mismatch between receiving funding and making this information available within CPGs, our understanding of general CPG funding practices is limited [5]. To assess the potential biases of the funding process, transparency is critical. In-depth knowledge of funding practices is also necessary to develop sustainable funding mechanisms to provide high quality guideline development in the future. The goal of this study, therefore, is to explore the accuracy and comprehensiveness of reporting on funding in German CPGs.

## Methods

We selected all finalised CPGs published on the homepage of the AWMF [2]. All valid CPGs available on 10th of July 2020 were included in the main analysis. There were no other inclusion or exclusion criteria. We screened publicly accessible guideline texts (short, long and patient versions, reports) for statements on CPG funding and filed them into a database.

Different appraisal tools for CPGs have been developed and are commonly used [15]. These tools cover a range of quality dimensions, such as the evaluation of evidence (grading of evidence, consistency between evidence and recommendations) or the consideration of different perspectives (e.g., patient perspectives) [15]. For analysis, we utilised the German Instrument for Methodological Guideline Appraisal Version: 2005/2006 + Domain 8 (2008) (DELBI) [16] and assessed the structural dimension “independence” (“Guideline development organization and funding”) using Criterion 22. We decided to use the DELBI instrument in favor of the more widely used AGREE-II tool [10] as both tools are considered to be equally adequate for comprehensive guideline appraisal [15]. However, the DELBI rating (1–4, from lowest to highest) can be made from the information provided by the guideline authors in the accessible CPG documentation (Table 1).

**Table 1:** DELBI Criterion 22 (“The guideline is editorially independent of the funding organisation(s).”)

Rating			Interpretation
“Strongly Disagree”	1	if the guideline does not contain any information / explanation pertaining to financial support.	if the CPG contained no statement or information provided was inconclusive.
	2	requires that the guideline provides information about both the responsible organisation (editors) and any additional financial or other support of the guideline.	if the CPG contained information on funding sources.
	3	requires that the guideline provides information about the funding or supporting organisation and details about how the guideline work has been funded.	if the CPG contained information on funding sources and expenses.
“Strongly Agree”	4	requires that apart from naming the funding and / or supporting bodies the guideline must contain information about the nature and volume of the funding. This should take the form of an explicit statement pointing out that no influence on the contents of the guideline has been exercised by the funding and/or supporting bodies. The information may be provided by the guideline itself or in a guideline report (see item 29). The guideline report must clearly be indicated in the guideline. According to these requirements the description in a guideline report must individually refer to the respective guideline; it is not sufficient to provide basic information only.	if the CPG contained information on funding sources, type and volume of expenses. The rating was also awarded if no exact costs were given for individual expenses (e.g. no information on the actual amount of travel costs). The information needed to relate to the guideline in question and to contain an explicit statement on the independence of all funding bodies and the responsible organization.

Table 1 DELBI criterion 22 (Domain 6: Editorial independence) was used [16]. The interpretation links the rating to the available information provided by the CPG authors (see Results section).

We explored the categories derived from the DELBI criterion 22 (Table 1) and developed further categories to determine funding sources, expenses and the amount of funding provided. Funding sources were categorised using examples provided by the AWMF [2]. This included own contributions by CPG authors (“honorary office”). In most cases, guideline authors are not reimbursed for their work (excluding e.g., travel costs for consensus meetings). Although one could argue that this is not comparable to the other funding categories, the AWMF classifies author’s voluntary contributions as a component of guideline funding. It is important to assess the role of guideline authors to distinguish between the potential influence of the funding bodies and individual COIs on guideline recommendations. Because the aim of this study was to explore the accuracy and comprehensiveness of reporting on funding, individual COIs were intentionally left out of this analysis. Indirect funding of CPG producing organisations was also not assessed.

All categories were piloted by two authors in a selected subset of CPGs. Two authors carried out the data extraction independently. A third author was consulted if consensus could not be reached. Reviewers were excluded from the extraction of specific CPGs if they took part during their development process. Funding information of CPGs was considered inconclusive if no direct connection could be made between funding sources and expenses. For example, the statement “There was no external funding for the guideline.” is ambigious as to whether the CPG was funded by the guideline producing organisation or there was no actual funding at all. CPGs with missing or inconclusive information were not excluded from the analysis to provide an overall picture of the transparency of CPG funding. These CPGs received a DELBI rating of 1 (Table 1).

An exploratory data analysis was performed using R [17, 18]. Counts and percentages were used to report the data extracted from the CPGs. The non-parametric Kruskal-Wallis test was used to assess differences on DELBI scores between the different guideline tiers. Pairwise comparisons were done by using post hoc Wilcoxon test with Bonferroni-Holm correction for multiple testing. We performed a sensitivity analysis with a full dataset including expired CPGs since they are accessible older CPGs may be less transparent with respect to their funding. The study used only information based on routine-data and was performed in accordance with the Helsinki declaration and the International Committee of Medical Journal Editors [19].

## Results

### CPG selection

We found 763 CPGs through our search in the AWMF registry. After de-duplication of one (017–49/053 − 012), 762 CPGs could be assessed. 507 guidelines (66.5%, S1 = 175, S2e = 27, S2k = 171, S3 = 134) were published in 2015 or later. The remaining, expired CPGs (n = 255) were developed between 2006 and 2015 (n = 10 between 2006 and 2010). The complete dataset can be found in the Appendix (**Additional file 1: Dataset**).

### Funding information provided by CPGs

**Fig. 1:**
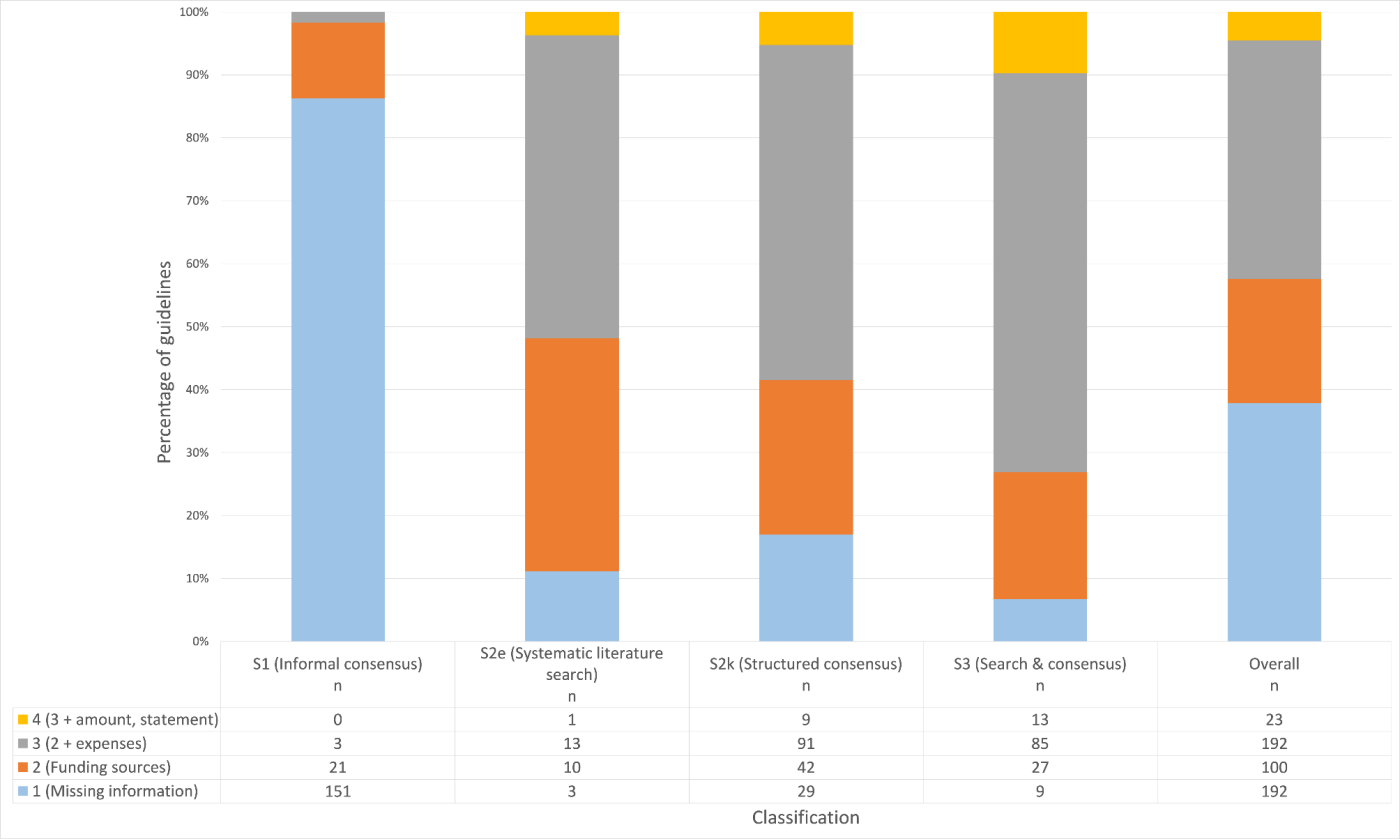
DELBI criterion 22 scores grouped by guideline grading

315/507 (62.1%) of all CPGs provided conclusive information on guideline funding (Table 2a). There were stark differences between the different guideline tiers. While S2k/e and S3 CPGs provided information in > 80% of cases, only around 15% of S1 CPGs included conclusive information. Of all CPGs containing a statement 215/507 (42.4%) included a statement on funding types as well. A trend towards more transparent reporting from S1 to S3 tier could be seen (Fig. 1). While 144/507 (28.4%) of all CPGs declared that they were independent of external funding, statements to this effect were only found in 3/175 (1.7%) of S1 CPGs.

**Table 2:** Funding of Clinical practice guidelines

2a Funding information provided by CPGs						
Classification		S1	S2e	S2k	S3	Overall
		Informal consensus	Systematic literature search	Structured consensus	Systematic literature search & Structured consensus	
n		175	27	171	134	507
Funding statement (%)	No	113 (64.6)	2 (7.4)	20 (11.7)	0 (0.0)	135 (26.6)
	Yes	24 (13.7)	24 (88.9)	142 (83.0)	125 (93.3)	315 (62.1)
	Inconclusive	38 (21.7)	1 (3.7)	9 (5.3)	9 (6.7)	57 (11.2)
Funding source mentioned (%)		24 (13.7)	24 (88.9)	142 (83.0)	125 (93.3)	315 (62.1)
Funding type mentioned (%)		3 (1.7)	14 (51.9)	100 (58.5)	98 (73.1)	215 (42.4)
Statement of independence (%)		3 (1.7)	14 (51.9)	51 (29.8)	76 (56.7)	144 (28.4)
**2b Funding sources for CPGs with available information**						
Classification		S1	S2e	S2k	S3	Overall
n		24	24	142	125	315
Scientific medical societies (%)		5 (20.8)	15 (62.5)	120 (84.5)	95 (76.0)	235 (74.6)
Contributions by authors (%)		22 (91.7)	14 (58.3)	68 (47.9)	66 (52.8)	170 (54.0)
Hospitals/Universities (%)		0 (0.0)	5 (20.8)	13 (9.2)	22 (17.6)	40 (12.7)
Independent agencies (%)		0 (0.0)	2 (8.3)	12 (8.5)	15 (12.0)	29 (9.2)
Guideline programs (%)		0 (0.0)	0 (0.0)	1 (0.7)	26 (20.8)	27 (8.6)
Self-regulatory bodies (%)		0 (0.0)	0 (0.0)	2 (1.4)	10 (8.0)	10 (3.2)
German Federal Government (%)		1 (4.2)	0 (0.0)	1 (0.7)	5 (4.0)	7 (2.2)
Pharmaceutical industry (%)		0 (0.0)	1 (4.2)	0 (0.0)	4 (3.2)	5 (1.6)
Other (%)		0 (0.0)	0 (0.0)	1 (0.7)	3 (2.4)	4 (1.3)
Insurances (%)		0 (0.0)	0 (0.0)	1 (0.7)	0 (0.0)	1 (0.3)
**2c Funding types for CPGs with available information**						
Classification		S1	S2e	S2k	S3	Overall
n		3	14	100	98	215
Meeting costs (%)		1 (33.3)	9 (64.2)	94 (94.0)	92 (93.9)	196 (91.2)
Scientific costs (%)		1 (33.3)	8 (57.1)	17 (17.0)	69 (70.4)	95 (44.2)
Administrative costs (%)		2 (66.6)	5 (35.7)	8 (8.0)	49 (50.0)	64 (29.8)
Material costs (%)		0 (0.0)	3 (21.4)	16 (16.0)	38 (38.8)	57 (26.5)
Independent literature search and appraisal (%)		0 (0.0)	0 (0.0)	3 (3.0)	10 (10.2)	13 (6.0)
Other (%)		0 (0.0)	1 (7.1)	0 (0.0)	2 (2.0)	3 (1.4)

**2 b** Personal contributions by authors: honorary offices by guideline authors, donations by guideline authors; Independent agencies: patient organizations, funding associations of charitable foundations; Guideline programs: German Program for National Clinical Practice Guidelines (NVL Program), Guideline Program in Oncology (OL program); Self-regulatory bodies: e.g. National Association of Statutory Health Insurance Physicians; Insurances: statutory health insurance, private health insurance; Other: AWMF (S3), Conference surpluses (S3), Südwestmetall (S2k), Wilhelm-Woort-Price 2016 (S2k); multiple answers per CPG possible **c** Scientific costs: methodology (e.g. literature searches, critical appraisal), implementation; Administrative costs: secretaries, infrastructure ; Material costs: databases, publication, layout, purchasing literature; Meeting costs: Travel expenses for consensus conferences and working sessions, facility costs, moderators; Other: Course fees (S3), External designer (S3), Naive Panel (S2e); multiple answers per CPG possible. Only valid CPGs are shown.

### DELBI criterion 22 by guideline tier

DELBI scores stratified by the guideline tiers are displayed in Table 2a and Fig. 1. Only 23/507 (4.5%) of the CPGs achieved the highest score. A total of 192/507 (37.9%) received the lowest rating (1 of 4) not reporting any (135/507, 26.6%) or inconclusive (57/507, 11.2%) funding information. This was mainly due to S1 guidelines (DELBI (1) = 151/175, 86.3%). No CPG in this tier achieved the highest score.

A Kruskal-Wallis test to examine the differences of the DELBI score showed significant differences (χ2 (3) = 264, n = 507, p = 6.17*10^− 53^) between the different guideline tiers. Pairwise comparisons indicated that S1 scores were observed to be significantly different from all other groups (S2e: p = 4.28*10^− 29^, S2k: p = 3.24*10^− 40^, S3: p = 4.02*10^− 47^) and that S2k scores were observed to be significantly different from the S3 group (p = 6*10^− 3^). The other differences between the scores (S2e/S2k, S2e/S3) were not statistically significant.

### Funding sources for CPGs with available information

We could assess funding sources of 315/507 (62.1%) CPGs (Table 2b). Scientific societies were most often (74.6%) mentioned with major differences between S1 (20.8%) and the other tiers (62.5–84.5%). Contributions by guideline authors were the second most common funding source mentioned overall. Other funding sources, such as hospitals and universities (as employers), independent foundations (e.g., charities, guideline programs (e.g., German Guideline Program in Oncology), self-regulatory bodies and the German Federal Government were almost exclusively mentioned in higher tiered CPGs. Funding through corporations in the healthcare sector (e.g., pharmaceutical industry, medical device manufacturers) was mentioned by five CPGs, once in an S2e and four times in S3 guidelines.

### Funding types for CPGs with available information

Funding types of 215/507 (42.4%) CPGs could be assessed (Table 2c). Meeting costs were mentioned in 196/215 (91.2%). Specific funding types were stated in S2k and S3 CPGs due to the need to conduct consensus conferences and the necessity of systematic literature appraisal. Independent methodological support for systematic literature appraisal through independent bodies (e.g., Institute for Quality and Efficiency in Health Care, IQWIG), an assumed quality measure, was mentioned in 13/215 6% (6%) of CPGs, mainly in S3 CPGs. Administrative costs weere only addressed in around 30% of CPGs although these are expected in all guideline tiers.

### Amount of funding provided for CPGs with available information

The amount of funding provided for CPG development was rarely documented and could therefore not be analysed. We could not establish whether the amount of external funding, or the different types of funding had an impact on the amount of funding. The available sums can be found in the raw dataset **(Additional file 1: Dataset**). However, these represent only approximations because commonly, only aggregate or indirect information was available (e.g., workload of people involved, paygrades).

### Sensitivity analysis

For the sensitivity analysis we also included expired CPGs. A total of 762 guidelines could be analysed (S1 = 283, S2e = 39, S2k = 249, S3 = 191). The analysis did not show any differences compared to the main analysis where expired CPGs were excluded (**Additional file 2: Sensitivity analysis**) indicating that the accuracy and completeness of reporting on funding of “out-of-date” CPGs is comparable to valid CPGs and there is no trend towards better reporting of newer CPGs.

## Discussion

### Main findings

This is the first structured description of the accuracy and comprehensiveness of reporting on funding within German clinical practice guidelines. It can thereby help to inform the public, guideline users, guideline producing scientific societies and funding bodies on strengths and shortcomings of funding practices as well as potential improvements. The funding processes of CPGs in Germany were not reported transparently as shown by rating all 507 valid AWMF guidelines with Criterion 22 of the DELBI tool. The guideline tier had an influence on the transparency scores and funding sources reported. The information presented here suggests that there is incomplete reporting of funding types.

### Context of other work

There is evidence from observational studies that individual COI are associated with prescribing patterns. A systematic review and meta-analysis by Brax et al. included 19 studies with different types of interactions, including detailing, industry-funded continuing medical education, and free gifts. They found moderate quality evidence that physicians’ interactions with pharmaceutical companies are associated with their prescribing patterns (OR = 2.52) [20]. A study among 41,257 French General Practitioners found better drug prescription efficiency indicators (e.g., prescription of generic drugs) and less costly drug prescriptions for GPs who did not receive gifts from pharmaceutical companies compared to GPs who received gifts [21].

A Cochrane review [7] that assessed the association of conflicts of interests in clinical guidelines, advisory committee reports, opinion pieces, and narrative reviews with recommendations supports these findings (RR: 1.26, 95% CI: 1.09 to 1.44). However, there was no significant difference for CPGs (4 studies with 86 CPGs, RR: 1.26, 95% CI: 0.93 to 1.69), probably related to the small sample size. Norris et al. included twelve studies that target the impact of COI on guideline quality [9, 22] with very limited evidence on the extent of commercial influence [23, 24].

There is also only limited information on the potential impact of guideline funding as shown by Campsall et al. (290 CPGs by 95 organizations) [13]. In this study, more comprehensive COI policies were associated with fewer positive and more negative recommendations regarding patented biomedical products. However, there were no disclosures for financial relationships of organizations although 63% (60/95) reported receiving funds from a biomedical company [13]. As shown above, Elder et al. [14] also revealed a mismatch between the organizational funding of medical societies from the industry and the disclosures in the CPGs. Besides non-transparent reporting, insufficient funding might also influence CPG quality.

Boyd et al. [6] recommend (1) public funding for guideline development, (2) development without commercial support, (3) no COI related to the subject matter and (4) no single source sponsorship. More transparent reporting might help to better understand funding mechanisms and costs of guideline development. This could inform policy makers on how to improve funding practices to ensure high quality CPG development and regulate funding mechanisms to enable timely updates in the future. The AWMF has called for sustainable and independent funding of high-quality guidelines [25]. Steps were already taken in Germany by implementing funding schemes for the systematic analysis of the scientific evidence through the *Innovationsfonds* (Innovation fund). In addition, independent guideline programs targeting oncology (Leitlinienprogramm Onkologie) and highly prevalent diseases (Nationale VersorgungsLeitlinien, NVL) exist. These programs should be further expanded and used as models for the implementation of other structured programs. However, independence of guideline development and recommendations always needs to be ensured.

### Limitations and strengths

A strength of our study is that it relied on a comprehensive listing of German guidelines, rather than a selected sample. However, we cannot generalise these results to guidelines not published in the registry but frequently used in the German speaking context (e.g., CPGs produced by the European Society of Cardiology, other international societies), or other healthcare systems (e.g., UK or USA). This study may not only raise awareness for more transparent reporting of funding practices but, more importantly, challenge the status quo and enable more sustainable and independent funding practices in the future. The development of categories to properly define funding expenses and interpretation of texts provided by the guideline authors was difficult. We neither assessed indirect funding of guideline developing organisations nor individual COI. Mandatory reporting with clear rules and templates, as in the handling of conflicts of interests, could overcome this limitation in future research.

### Policy implications

Transparency of guideline funding could be achieved by making it mandatory to publish information for all guidelines. For that purpose, a standardised form and guidance should be developed by the AWMF. This should contain information on the funding bodies and their COI related to the subject matter (e.g., grants from the pharmaceutical industry towards scientific medical societies) [6], expenses and the amount of funding. However, we need to find the right balance between the need for transparency and feasibility. In our own experience, guideline development is complex and, in most cases, the exact costs of an individual’s contribution cannot be calculated. The benefits from publishing details on guideline funding must therefore outweigh the bureaucratic burden. All information should relate to the specific guideline and contain an explicit statement on the independence of the body that develops the guideline from all funding bodies [16]. In case of CPGs exclusively or primarily developed through the contributions of the guideline authors, individual conflicts of interests play a big role. Therefore, funding practices and individual COIs both need to be assessed to get a complete picture.

## Conclusion

In recent years, steps were taken in the right direction towards more transparent reporting and handling of potential conflicts of interests by guideline authors in Germany [26]. In the context of guideline funding, this process is still in its infancy. The development of standard forms – as already used for reporting of individual COI - could enhance transparent reporting of funding mechanisms and thereby trust in clinical practice guidelines. Germany could take a proactive role globally and thereby influence other countries to take the same steps.

## Electronic supplementary material

Below is the link to the electronic supplementary material.

**Additional File 1**: Dataset

**Additional File 2**: Sensitivity analysis

**Additional File 3**: Additional information for the dataset

## Data Availability

All data generated or analysed during this study are included in this published article [and its supplementary information files]. The dataset (**Additional file 1: Dataset)** can be accessed in a non-proprietary format. The dataset includes funding information and DELBI ratings for all CPGs that were analysed in this study.

## Abbreviations

AGREE-II: Appraisal of Guidelines for Research & Evaluation Instrument
AWMF: Association of Scientific Medical Societies in Germany (Arbeitsgemeinschaft der Wissenschaftlichen Medizinischen Fachgesellschaften)
COI: Conflict(s) of interest
CPG: Clinical practice guidelines
DELBI: German Instrument for Methodological Guideline Appraisal (Deutsches Leitlinien-Bewertungsinstrument)
