# Supplementary material for: Transparency of clinical practice guideline funding: a cross-sectional analysis of the German AWMF registry
Source: BMC Med Ethics. 2023 May 19;24:32. doi: 10.1186/s12910-023-00913-0 (PMC10199475; doi:10.1186/s12910-023-00913-0)
Supplement: Supplementary file 1 — Additional File 1: Dataset [file 12910_2023_913_MOESM1_ESM.docx]

Additional file 2: Sensitivity analysis for the full-analysis set (including expired CPGs)

| **e1a** Funding information provided by CPGs | | | | | | |
| --- | --- | --- | --- | --- | --- | --- |
| Classification |  | S1 | S2e | S2k | S3 | Overall |
|  |  | Informal consensus | Systematic literature search | Structured consensus | Systematic literature search & Structured consensus |  |
| n |  | 283 | 39 | 249 | 191 | 762 |
| Funding statement (%) | No | 193 (68.2) | 2 (5.1) | 35 (14.1) | 2 (1.0) | 232 (30.4) |
|  | Yes | 45 (15.9) | 36 (92.3) | 203 (81.5) | 178 (93.2) | 462 (60.6) |
|  | Inconclusive | 45 (15.9) | 1 (2.6) | 11 (4.4) | 11 (5.8) | 68 (8.9) |
| Funding source mentioned (%) | | 45 (15.9) | 36 (92.3) | 203 (81.5) | 178 (93.2) | 462 (60.6) |
| Funding type mentioned (%) | | 11 (3.9) | 20 (51.2) | 143 (57.4) | 145 (75.9) | 319 (41.9) |
| Statement of independency (%) | | 7 (2.5) | 18 (46.2) | 72 (28.9) | 101 (52.9) | 198 (26.0) |
| DELBI Criterion 22 (%) | 1 | 238 (84.1) | 3 ( 7.7) | 46 (18.5) | 13 (6.8) | 300 (39.4) |
|  | 2 | 34 (12.0) | 16 (41.0) | 60 (24.1) | 33 (17.3) | 143 (18.8) |
|  | 3 | 11 (3.9) | 18 (46.2) | 133 (53.4) | 130 (68.1) | 292 (38.3) |
|  | 4 | 0 (0.0) | 2 (5.1) | 10 (4.0) | 15 (7.9) | 27 (3.5) |
| **e1b** Funding sources for CPGs with available information | | | | | | |
| Classification | | S1 | S2e | S2k | S3 | Overall |
| n | | 45 | 36 | 203 | 178 | 462 |
| Scientific medical societies (%) | | 14 (31.1) | 24 (66.7) | 166 (81.8) | 139 (78.1) | 343 (74.2) |
| Contributions by authors (%) | | 39 (86.7) | 20 (55.6) | 106 (52.2) | 100 (56.2) | 265 (57.4) |
| Hospitals/Universities (%) | | 2 (4.4) | 5 (13.9) | 20 (9.9) | 32 (18.0) | 59 (12.8) |
| Independent agencies (%) | | 0 (0.0) | 3 (8.3) | 26 (12.8) | 21 (11.8) | 50 (10.8) |
| Guideline programs (%) | | 0 (0.0) | 0 (0.0) | 1 (0.5) | 37 (20.8) | 38 (8.2) |
| Self-regulatory bodies (%) | | 0 (0.0) | 0 (0.0) | 4 (2.0) | 10 (5.6) | 14 (3.0) |
| German Federal Government (%) | | 1 (2.2) | 0 ( 0.0) | 1 (0.5) | 7 (3.9) | 9 (1.9) |
| Pharmaceutical industry (%) | | 0 (0.0) | 1 (2.8) | 0 (0.0) | 4 (2.2) | 5 (1.1) |
| Other (%) | | 0 (0.0) | 0 (0.0) | 2 (0.9) | 2 (1.1) | 4 (0.9) |
| Insurances (%) | | 0 (0.0) | 0 (0.0) | 1 (0.5) | 2 (1.1) | 3 (0.6) |
|  | |  |  |  |  |  |
| **e1c** Funding types for CPGs with available information | | | | | | |
| Classification | | S1 | S2e | S2k | S3 | Overall |
| n | | 11 | 20 | 143 | 145 | 319 |
| Meeting costs (%) | | 7 (63.6) | 14 (70.0) | 135 (94.4) | 134 (92.4) | 290 (90.9) |
| Scientific costs (%) | | 1 (9.1) | 9 (45.0) | 23 (16.1) | 100 (69.0) | 133 (41.7) |
| Administrative costs (%) | | 3 (27.3) | 6 (30.0) | 12 (8.4) | 77 (53.1) | 98 (30.7) |
| Material costs (%) | | 1 (9.1) | 3 (15.0) | 18 (12.6) | 60 (41.4) | 82 (25.7) |
| Independent literature search and appraisal (%) | | 0 (0.0) | 0 (0.0) | 3 (2.1) | 11 (7.6) | 14 (4.4) |
| Other (%) |  | 0 (0.0) | 1 (5.0) | 0 (0.0) | 2 (1.4) | 3 (0.9) |
| **e1 b** Personal contributions by authors: honorary offices by guideline authors, donations by guideline authors; Independent agencies: patient organizations, funding associations of charitable foundations; Guideline programs: German Program for National Clinical Practice Guidelines (NVL Program), Guideline Program in Oncology (OL program); Self-regulatory bodies: e.g. National Association of Statutory Health Insurance Physicians; Insurances: statutory health insurance, private health insurance; Other: AWMF (S3), Conference surpluses (S3), Südwestmetall (S2k), Wilhelm-Woort-Price 2016 (S2k); multiple answers per CPG possible **c** Scientific costs: methodology (e.g. literature searches, critical appraisal), implementation; Administrative costs: secretaries, infrastructure ; Material costs: databases, publication, layout, purchasing literature; Meeting costs: Travel expenses for consensus conferences and working sessions, facility costs, moderators; Other: Course fees (S3), External designer (S3), Naive Panel (S2e); multiple answers per CPG possible. | | | | | | |
