# Supplementary material for: Transparency of clinical practice guideline funding: a cross-sectional analysis of the German AWMF registry
Source: BMC Med Ethics. 2023 May 19;24:32. doi: 10.1186/s12910-023-00913-0 (PMC10199475; doi:10.1186/s12910-023-00913-0)
Supplement: Supplementary file 3 — Additional File 3: Additional information for the dataset [file 12910_2023_913_MOESM3_ESM.docx]

Additional information for the dataset for the article Transparency of clinical practice guideline funding: A cross-sectional analysis of the German AWMF registry by Napierala et al., BMC Medical Ethics

Legend: This dataset accompanies the article *Transparency of clinical practice guideline funding: A cross-sectional analysis of the German AWMF registry by Napierala et al., published in BMC Medical Ethics* and provides all necessary data for the analyses in the results section. The dataset is provided in a proprietary format (csv). Explanations: First Reviewer (HN: Hendrik Napierala), Second Reviewer (AS: Angela Schuster, SGB: Sabine Gehrke-Beck, CH: Christoph Heintze), ID: Identifier in the dataset, ID in the AWMF registry: Unique identifier for each guideline in the registry of the Association of the Scientific Medical Societies in Germany, Classification: S1, S2e, S2k, S3, as defined by the AWMF, Valid until (YYMM): The expiry date of each guideline is provided in the YYMM format, the other variables can take the following values: Funding statement provided (Yes/No/Inconclusive), Report individually refers to the respective guideline (Yes/No), Funding type mentioned (Yes/No), Funding type: scientific costs (Yes/No), Funding type: administrative costs (Yes/No), Funding type: material costs (Yes/No), Funding type: consensus conferences (Yes/No), Funding type: other (Yes/No), Funding type: external methodology (Yes/No), Funding source mentioned (Yes/No), Funding source: (Yes/No) authors (Yes/No), Funding source: authors sum (in €), Funding source: scientific society (Yes/No), Funding source: scientific society sum (in €), Funding source: independent agencies (Yes/No), Funding source: independent agencies sum (in €), Funding source: employers (Yes/No), Funding source: employers sum (in €), Funding source: guideline programme (Yes/No), Funding source: guideline programme sum (in €), Funding source: state funding (Yes/No), Funding source: state funding sum (in €), Funding source industry (Yes/No), Funding source: industry sum (in €), Funding source: self-government (Yes/No), Funding source: self-government sum (in €), Funding source: insurance (Yes/No), Funding source: insurance sum (in €), Funding source: other, Funding source: other sum (in €), Statement of independency (Yes/No), DELBI rating (1 (lowest) – 4 (highest))
